# Laser-Assisted In Situ Keratomileusis Surgery on a Patient with Myelin Oligodendrocyte Glycoprotein Antibody-Associated Disease or Neuromyelitis Optica Spectrum Disorder

**DOI:** 10.1155/2023/9977513

**Published:** 2023-08-24

**Authors:** Majid Moshirfar, Duncan J. Williams, Melody Ziari, Meagan D. Seay, Yasmyne C. Ronquillo

**Affiliations:** ^1^Hoopes Vision Research Center, Hoopes Vision, 11820 S. State St. #200, Draper, UT, USA 84020; ^2^John A. Moran Eye Center, University of Utah School of Medicine, Salt Lake City, UT, USA; ^3^Utah Lions Eye Bank, Murray, UT, USA; ^4^Arizona College of Osteopathic Medicine, Midwestern University, Glendale, AZ, USA; ^5^University of Texas Health Science Center at Houston, McGovern Medical School, Houston, TX, USA

## Abstract

Myelin oligodendrocyte glycoprotein antibody-associated disease (MOGAD) and neuromyelitis optica spectrum disorder (NMOSD) are rare central demyelinating diseases that may affect refractive surgery outcomes. Optic neuritis and brainstem syndromes affecting cranial nerves are particularly relevant to corneal refractive surgery (CRS), such as laser-assisted in situ keratomileusis (LASIK), photorefractive keratectomy, or small incision lenticule extraction. There is currently no existing literature concerning the outcomes of CRS in patients with MOGAD or NMOSD. This article reports the clinical outcome of a MOGAD patient who underwent LASIK.

## 1. Introduction

Myelin oligodendrocyte glycoprotein antibody-associated disease (MOGAD) and neuromyelitis optica spectrum disorder (NMOSD) are autoantibody-mediated diseases of the central nervous system. For decades, they were considered severe forms of multiple sclerosis (MS), but researchers recently delineated specific antibodies (Ab) that target myelin oligodendrocyte glycoprotein (MOG) or aquaporin-4 (AQP4) in the spinal cord, optic nerve, dorsal medulla, thalamus/hypothalamus, and brainstem [1, 2]. While MOGAD often meets the clinical criteria for NMOSD, defined as longitudinally extensive transverse myelitis, optic neuritis (ON), area postrema syndrome, and diencephalic, cerebral, or symptomatic brainstem syndromes [1, 2], MOGAD requires MOG-Ab seropositivity for diagnosis [2]. Studies examining laser-assisted in situ keratomileusis (LASIK) and photorefractive keratectomy (PRK) in patients with a history of MS-associated optic neuritis suggest that the procedure is safe [3]. However, little is reported about the safety and efficacy of refractive surgery in MOGAD or NMOSD patients. This article presents a case of successful LASIK surgery in a patient with MOGAD.

## 2. Case Report

A 28-year-old female with MOGAD presented for corneal refractive surgery (CRS) consultation. She was diagnosed with MOG-Ab seropositivity three years prior and was well controlled on rituximab 1 g infusion every 6 months. She was asymptomatic apart from ocular dryness that was well controlled with artificial tears. On examination, nystagmus was noted with rightward gaze worse than leftward gaze. Marcus Gunn testing showed no relative afferent pupillary defect. Slit lamp and dilated fundus examinations were unremarkable, and the visual fields were full to confrontation OU. Assessments of contrast sensitivity, corneal sensation, and color vision were within normal limits. Schirmer's testing without anesthesia over 5 minutes was 27 mm OD and 30 mm OS. Best-corrected distance visual acuity was 20/20 OU, and manifest refraction was -4.00 OD and −4.75 − 0.25 × 178 OS. After discussing the risks and benefits of this elective procedure and consultation with the patient's neuro-ophthalmologist and neurologist, femtosecond-assisted LASIK was performed without complications. Her postoperative course was uneventful, without relapse of MOGAD symptoms, alterations in contrast sensitivity or corneal surface dryness, or postoperative infectious keratitis. Her last uncorrected distance visual acuity was 20/20 OU at 6 months postoperatively, and the patient continues to follow-up with her neurologist and eye care provider.

## 3. Discussion

The prevalence of MOGAD is approximately 1.6 per million persons, whereas NMOSD ranges from 0.5 to 10 per 100,000 persons [2]. While MOGAD shows a gender ratio around 1 : 1 and is more common in children [2], NMOSD shows a strong predilection for females (3-9 : 1 ratio) [1] and increased prevalence and severity in East Asians and Blacks [1, 2]. Both diseases typically present in patients between 30 and 40 years of age [1, 2]. Despite the rarity of these diseases, corneal refractive surgeons should understand the necessary workup, clinical considerations, and contraindications to surgery.

A thorough history is critical as these diseases differ significantly in epidemiology, characteristic manifestations, and prognosis (Table 1). It is important to review cranial magnetic resonance imaging and optic nerve optical coherence tomography to determine the extent of baseline optic nerve damage and to preoperatively evaluate corneal sensation, color plates, automated visual field, and corneal dryness (Table 2).

Minor ocular trauma has been shown to trigger the onset of NMOSD and ON [4]; consequently, LASIK and PRK may carry this risk. Each attack of ON risks persistent (or permanent) loss of contrast sensitivity, color vision, and visual fields [3]. Therefore, we suggest that practitioners evaluate the baseline level of contrast sensitivity in MOGAD and NMOSD patients and utilize wavefront-guided technology [5] or customized topography laser treatments [6] that have been shown to minimize reductions in contrast sensitivity in the general population. While AQP4-Abs commonly involve relapsing disease with progressive vision loss [1, 2], adult patients with MOGAD generally present with relapsing ON with better visual recovery or monophasic disease altogether [1, 2]. Thus, CRS may be relatively safer and have prolonged visual benefits for MOGAD patients.

Beyond optic nerve involvement, MOGAD and NMOSD patients may have cranial nerve deficits, neuropathic pain, and oculomotor dysfunction [1, 2]. The presumed disruption of the corneal nerve plexus during LASIK and PRK may exacerbate ocular surface dryness and impede corneal wound healing [7] in these patients, who carry an elevated risk of cranial nerve deficits that could affect ocular surface homeostasis. Considering that over 83% of NMOSD and MOGAD patients suffer from neuropathic pain [2], the risk of postoperative corneal neuropathic pain may increase. Thus, we recommend a thorough investigation of ocular surface integrity and cranial nerve deficits preoperatively (Table 2). Regarding nystagmus, we suggest utilizing advanced ocular tracking platforms shown to be effective for existing excimer lasers [8].

Treatment for MOGAD and NMOSD typically involves intensive steroid use and chronic immunomodulating therapy for relapsing disease [1, 2]. Realizing that high-dose steroid use can transiently increase intraocular pressure and cause refractive instability [9], we recommend waiting for an extended period after acute therapy to allow for accurate evaluation of stable refractive error preoperatively. Due to the high relapse rate, especially with AQP4-Ab-positive NMOSD, these patients may be on long-term immunosuppressive agents [1, 2] that can increase the risk of viral and bacterial keratitis [10]. Although maintenance therapy reduces the number of relapses and improves overall prognosis, progressive deterioration may still occur [2].

In conclusion, significant visual impairment is a common finding in this population, and patients may seek refractive surgery to improve unaided visual acuity and decrease dependence on glasses and contact lenses. For those with strong visual recovery, as seen in our MOGAD patient, LASIK may be a successful option. We recommend a thorough preoperative workup assessing ocular surface integrity and visual function, along with a neurology consult. Candidates for CRS must be fully informed and understand that MOGAD and NMOSD carry the risk of ON with impaired vision and contrast sensitivity, cranial nerve deficits, and certain postoperative complications seen in immunocompromised patients. As minimal ocular trauma could potentially induce ON attacks, we advise extreme caution when considering CRS in patients with active or relapsing disease, especially those with AQP4-Abs. These recommendations are based on the most common clinical findings of MOGAD and NMOSD; however, given the wide spectrum of possible presentations, every patient should be individually evaluated by their ophthalmologist and care team.

## Figures and Tables

**Table 1:** Neuromyelitis optica spectrum disorder, further broken down into AQP4-seropositive and seronegative NMOSD, and myelin oligodendrocyte glycoprotein antibody-associated disease epidemiology, characteristic manifestations, and prognosis with risk factors for more severe disease.

	Epidemiology	Clinical findings	Prognosis
NMOSD	1 per 100,000 Caucasians^2^ 3.5-4.1 per 100,000 Asians^2^ 1.8-10 per 100,000 Blacks^1,2^ Onset 30-40 years^1,2^ Onset 28-33 years for Blacks^2^ Onset 35-40 years for Asians^2^ Onset 44 years for Caucasians^2^ 3 : 1-9 : 1 female/male^1^	LETM^1,2^ ON^1,2^ Area postrema syndrome^1,2^ Diencephalic syndromes^1,2^ Cerebral syndromes^1,2^ Brainstem syndromes^1,2^	Negative impact on quality of life:^2^ depression, neuropathic pain, bowel/bladder dysfunction, sexual dysfunction, inability to work, cognitive impairment Within 5 years untreated:^1^ 50% blind and wheelchair-bound, 33% will have died Better recovery:^2^ young adults, early treatment (<14 days) Greater recurrence/disability/death:^2^ age >60, age <12, Asian, Black, longer length of myelitis lesions, ON at disease onset, increased severity of initial attack 5 : 1-10 : 1 female/male recurrence^2^

AQP4-seropositive NMOSD	1/4 have coexistent autoimmune disease^1,2^ Uncommon in children^2^	Inflammation of posterior optic nerve segments (optic tracts & chiasm)^1,2^ Unilateral/chiasmal ON involving >1/2 optic nerve^2^ Myelitis involving >3 vertebral segments in 85%, especially cervical and thoracic cords^1,2^	Relapses in 90% involving ON and/or LETM^2^ Annual relapse rate 0.77 despite therapy^2^ More visual & motor disability^2^

Seronegative NMOSD	Rare^2^		More disability than MOGAD^2^

MOG-seropositive (MOGAD)	Onset mid-30 s^2^ 1 : 1 female/male^2^ 1.6/million overall^2^ 1.3/million adults^2^ 3.1/million children^2^ More common in children & elderly^1,2^ No racial preponderance^2^	Acute disseminated encephalomyelitis for age <7^2^ ON for age >7^2^ Inflammation of anterior optic nerve segments with increased edema^2^ Bilateral ON more common^2^ Lumbosacral myelitis^2^ Myelitis <3 vertebral segments^2^	Better visual recovery from ON^1,2^ Better motor recovery^2^ Relapses in 44-83%^2^ Can be monophasic without relapse^1,2^ 58% have residual disability^2^

Abbreviations: NMOSD: neuromyelitis optic spectrum disorder; LETM: longitudinally extensive transverse myelitis; ON: optic neuritis; AQP4: aquaporin-4; MOG: myelin oligodendrocyte glycoprotein; MOGAD: myelin oligodendrocyte glycoprotein antibody-associated disease.

**Table 2:** Suggested questions and testing for patients with neuromyelitis optica spectrum disorder and myelin oligodendrocyte glycoprotein antibody-associated disease to include in a thorough ophthalmologic evaluation.

Patient history	Do you have any history of eye pain? Have you ever been diagnosed with optic neuritis? How many recurrences of optic neuritis, if any? When was the last episode? Has anything triggered an episode (e.g., severe allergic reaction, surgery, trauma, etc.)? Are you currently on steroids or any long-term immunosuppressive agents? Do you have any history of dry eyes or recurrent corneal erosions? Do you have any history of eye infections (especially bacterial or viral corneal infection)? Have you ever had an episode of facial palsy, double vision, or nystagmus? Do you have any lasting visual deficits? Do you have neuropathic pain?
Testing	
Imaging	Review MRI brain/orbits with contrast to determine the extent of optic nerve involvement. Chiasmal involvement, longitudinally extensive optic nerve enhancement, and/or bilateral optic nerve involvement point to ON of NMOSD or MOGAD versus MS-associated ON.^1,2^ Review OCT optic nerve to determine the extent of optic nerve damage. Markedly reduced peripapillary nerve fiber layer thickness is commonly seen following ON of NMOSD and MOGAD.^1,2^
Current visual quality	Comprehensive dilated ophthalmic exam Pelli-Robson contrast sensitivity chart Red desaturation test, Ishihara color plate Automated perimetry testing
Ocular surface integrity	Schirmer testing, fluorescein dye, tear meniscus height, tear film analysis Corneal nerve sensation
Other	Determine the seropositivity with a cell-based assay to detect AQP4-IgG or MOG-IgG in serum. Titers do not reliably predict remission.^2^ Spontaneous nystagmus testing Comprehensive neurologic exam to determine the extent of neurologic deficits (especially cranial nerves 2, 3, 5, 7)

Abbreviations: MRI: magnetic resonance imaging; ON: optic neuritis; NMOSD: neuromyelitis optica spectrum disorder; MOGAD: myelin oligodendrocyte glycoprotein antibody-associated disease; MS: multiple sclerosis; OCT: optical coherence tomography; AQP4: aquaporin-4; IgG: immunoglobulin G; MOG: myelin oligodendrocyte glycoprotein.

## Data Availability

Data sharing is not applicable to this article as no datasets were generated or analyzed during the current study.

## Additional Points

*Publication Originality Statement.* We confirm that this publication is original. *Open Access.* This article is distributed under the terms of the Creative Commons Attribution Non-Commercial 4.0 International License (http://creativecommons.org/licenses/by-nc/4.0/), which permits any noncommercial use, distribution, and reproduction in any medium, provided you give appropriate credit to the original authors and the source, provide a link to the Creative Commons license, and indicate if changes were made.
